# Editorial: Targeting Estrogens in Cancer Care

**DOI:** 10.3389/fonc.2022.935938

**Published:** 2022-07-11

**Authors:** Marzia Di Donato

**Affiliations:** Department of Precision Medicine, University of Studies of Campania “L.Vanvitelli”, Naples, Italy

**Keywords:** estrogens, estrogen receptor, breast cancer, thyroid cancer, pan cancer analysis, adipokines, obesity-related tumors

Estrogens are pleiotropic female steroid hormones well recognized to be important in many physiological processes including sexual development and reproductive health. Unfortunately, they have also a key role in the development and progression of cancer. It is known that some types of cancer, including breast, ovarian and uterine cancers, are closely related to estrogenic action due to their progression (1). In addition, estrogens could influence the progression and aggressiveness of other types of cancer [e.g. melanoma (2), colon (3), prostate (4) (Di Zazzo et al.), pancreatic (5) and lung (6)], binding to Estrogen Receptors (ERα or ERβ) and acting through genomic (7–9) or non-genomic actions (9, 10). This Research Topic is focused on the role of Estrogens on Breast cancer (BC) cells but looks also at the effects of estrogen on Thyroid cancer (TC), at its relationship with adipokines in obesity-related tumors and at the end makes an overview on the analysis of *ESR1*, *ESR2*, and *PGR genes* in different types of cancer. Estrogens and more in general Steroid hormones (SHs) have a great impact on cancerogenesis. In this context, in their review, Saha et al. point to analyze the role of SHs in BC. Firstly, they present in detail the non-genomic and genomic actions of SHs in BC. Then, they describe the role of SHs in all the phases of cell cycle, lingering in cell cycle anomalies exerted by SHs in BC development and progression. The authors describe the role of the cell cycle inhibitors used in clinical trials for the management of patients affected by BC. They conclude observing that different cyclin-dependent kinase (CDK) inhibitors, in particular CDK4/6, have emerged as novel therapeutical approaches to be used as mono- or combinatorial use with SHR-related therapeutics for a better management of BC patients.

Estrogens and ERα can contribute to the radioresistance and chemoresistance in BC. Particular attention to this topic is given by Jiménez-Salazar et al. Their review is focused on the role of the non-genomic actions of estrogens in the process of the DNA repair and related activated pathways (e.g., c-Src, EGFR, HER2, ERK, or PI3K/AKT), and the relationship occurring with chemoresistance. They analyze point by point the role of estrogen and ERα in chemoresistance which follows to the alteration of DNA Repair mechanisms, the prevention of apoptosis, and/or deregulation of the cell cycle occurring in breast cancerous cells. Estrogens have also an important role in obesity-related tumors. As reported by Pu and Chen, people affected by obesity show high circulating levels of estrogen, which can promote the deposition and function of adipose tissue. The authors analyze the link between adiponectin, leptin, insulin, and estrogen that are all secreted by tumor microenvironment cells surrounding obesity-related tumors (e.g, breast cancer, pancreatic cancer, ovarian cancer, and colorectal cancer). Furthermore, the authors describe the role of other three adipokines [resistin, Monocyte Chemoattractant Protein-1 (MCP-1) and Macrophage Migration Inhibitory Factor (MIF-1)] in cancer. Finally, they emphasize the importance of new therapeutic strategies, that targeting the signaling circuits activated by these molecules, could be useful for the treatment of obesity-related tumors.

Ding and Kuang identify, in their manuscript, the Haem-oxidized IRP2 Ubiquitin Ligase-1 (HOIL-1) as a positive modulator of ERα in BC. HOIL-1, which is highly expressed in human BC derived samples, results in a ubiquitin ligase able to modulate ERα protein stability. In the extra nuclear compartment of the cells (cytoplasm), HOIL-1, complexing with the AF1 domain of ERα, through its RING domain, prolongs ERα protein stability, inducing BC cell proliferation. Instead, in the nuclear compartment of the cells, HOIL-1 co-activates ERα gene expression. Thus, HOIL-1 has a role in both genomic and post-translational regulation of ERα signaling pathway.

Another important marker in BC seems to be HOXA1. When it is *de novo* expressed or overexpressed represents a marker of cancer progression or poor prognosis. Through a Bioinformatic analysis of genome-wide mRNA expression of human BC derived samples Belpaire et al. show that HOXA1 mRNA expression is higher in more malignant BC subtypes, supporting the role of HOXA1 in cancer aggressiveness. The authors describe an inverse correlation between genes associated with HOXA1 expression and the ER status. HOXA1 can inhibit ERα activity when its DNA-binding homeodomain is intact. HOXA1 and ERα can also physically interact in the nuclear compartment of the cells but without resulting essential for ERα transcription activity inhibition by HOXA1. In conclusion, while HOXA1 can inhibit ERα activity, ERα cannot repress HOXA1 function.

In addition to BC, as mentioned before, ER α and β have also a role in other types of cancer. Shen et al. analyze the expression of *ESR1*, *ESR2* and *PGR* by using the Gene Expression Profiling Interactive Analysis 2 (GEPIA2) and cBioPortal in a comprehensive pan-cancer analysis. They investigate the ERα, ERβ and Progesterone Receptor (PgR) mRNA expression, the genetic alternations, and the clinical outcomes as well as the co-expression of their genes with immunomodulatory factors in a variety of cancer types. By using the TISIDB database, the authors observe a correlation between *ESR1*, the immunoinhibitors (CD274, CD96, CFS1R, and CTLA-4) and the immunostimulators (such as CD27, CD28, and CXCL12) opening new frontiers in the immunotherapy related to SHs receptors. Then, they correlate the genes expression with the overall survival (OS) and disease-free survival (RFS) in patients, confirming the role of these genes as prognostic markers and therapeutic targets for multiple cancers.

Lastly, Liu et al. focus their attention on the role of ER in TC. TC cells express both ERα and ERβ also if at different extent. ERα can modulate the cell proliferation of TC cells, while ERβ or the use of its agonists can inhibit the proliferation of TC cells. In their nice review, the authors analyze the molecular mechanism activated by ER and the signaling effectors involved in TC cells with particular attention to PI3K/Akt axis, MAPK cascade, reactive oxygen species (ROS) related pathways. In addition, they also describe the role of Estrogen-Mediated Signaling Pathways in TC Tumor Microenvironment (VEGF signaling, HIF-1 role, NF-kb pathway). Finally, they observe that occurs a crosstalk among all the mentioned cascade circuits.

This Research Topic is interesting and contains some notable ideas. It further clarifies the need to find other pathways, which by interconnecting with ER signaling might result, if targeted, useful in the fight against cancer.

## Author Contributions

MDD wrote, read and approved the submitted version.

## Conflict of Interest

The author declares that the research was conducted in the absence of any commercial or financial relationships that could be construed as a potential conflict of interest.

## Publisher’s Note

All claims expressed in this article are solely those of the authors and do not necessarily represent those of their affiliated organizations, or those of the publisher, the editors and the reviewers. Any product that may be evaluated in this article, or claim that may be made by its manufacturer, is not guaranteed or endorsed by the publisher.
